# Effect of storage and preconditioning of healing rat Achilles tendon on structural and mechanical properties

**DOI:** 10.1038/s41598-020-80299-w

**Published:** 2021-01-13

**Authors:** Franciele Dietrich-Zagonel, Malin Hammerman, Magnus Bernhardsson, Pernilla Eliasson

**Affiliations:** 1 Orthopedics, Department of Biomedical and Clinical Sciences, Division of Surgery, Orthopedics and Oncology, Faculty of Medicine and Health Science, Linköping University, 581-83 Linköping, Sweden; 2 Department of Biomedical Engineering, Lund University, 221-00 Lund, Sweden

**Keywords:** Musculoskeletal system, Mechanical properties

## Abstract

Tendon tissue storage and preconditioning are often used in biomechanical experiments and whether this generates alterations in tissue properties is essential to know. The effect of storage and preconditioning on dense connective tissues, like tendons, is fairly understood. However, healing tendons are unlike and contain a loose connective tissue. Therefore, we investigated if storage of healing tendons in the fridge or freezer changed the mechanical properties compared to fresh tendons, using a pull-to-failure or a creep test. Tissue morphology and cell viability were also evaluated. Additionally, two preconditioning levels were tested. Rats underwent Achilles tendon transection and were euthanized 12 days postoperatively. Statistical analyzes were done with one-way ANOVA or Student’s t-test. Tissue force and stress were unaltered by storage and preconditioning compared to fresh samples, while high preconditioning increased the stiffness and modulus (p ≤ 0.007). Furthermore, both storage conditions did not modify the viscoelastic properties of the healing tendon, but altered transverse area, gap length, and water content. Cell viability was reduced after freezing. In conclusion, preconditioning on healing tissues can introduce mechanical data bias when having extensive tissue strength diversity. Storage can be used before biomechanical testing if structural properties are measured on the day of testing.

## Introduction

Biomechanical tests of various tissues are an essential part of experimental research to understand and improve connective tissue biology. However, these tests are not always possible to conduct straight after tissue collection, as transportation to test facilities or multiple collection time-points are sometimes needed. Therefore, storage of the samples at different temperatures (e.g. at 8 °C in the fridge or at − 18 °C in the freezer) is recurrently required. Tendon research often uses animal models to study the development of tendon healing and mechanical parameters are frequently the main outcomes^1–4^. Tendons heal through formation of a fibrovascular scar tissue by an orchestra of different biological mechanisms and the material and structural properties of the newly formed tissue are important to verify its function^1,2,5^. Hence, it is fundamental to understand how storage can affect various tissue properties. The effect of storage has been studied on intact tendons but not on healing tendons to our knowledge. Previous studies on intact tendons have somewhat diverging results. Freezing has been shown to alter some structural and mechanical parameters such as ultimate force, stress and modulus^6,7^. On the other hand, another study has shown that freezing or repeatedly freeze-thawing cycles did not affect intact tendons even on microscale level^8^. Although damage in the interfibrillar space was observed, the load-bearing collagen was not affected^8^.

Precondition of tendon specimens prior to biomechanical testing is a common laboratory routine, in order to normalize the load history of unequal samples^9^. This technique has been shown to influence the collagen fiber re-alignment in the intact tendon^10^. However, the advantage of preconditioning on intact tendons might not be the same for healing tendons as the tissue composition differs. Healing tendons are often weaker, contain more water and proteoglycans, are less stiff, and have more viscoelastic behavior^4,11^. Twelve days of healing in rat Achilles tendon corresponds to the proliferatory/early remodeling phase^2^ and samples collected at this stage display a large callus tissue that is hypercellular and contain disorganized collagen fibers^12^. Moreover, the healing tendons also comprise two types of tissue with different material properties; a dense intact/old tendon stump and a loose newly formed tissue^2,13^. Less is known about how preconditioning influences this newly formed connective tissue. This knowledge is an important issue for future studies and approaches.

Therefore, the objective of this study was to investigate if the elastic and viscoelastic properties of healing rat Achilles tendons were altered by short-term storage in the fridge or freezer. Furthermore, we also aimed to investigate how different levels of preconditioning influenced the mechanical properties of the healing Achilles tendons.

## Results

### The effect of storage in a pull-to-failure test (experiment 1)

No significant differences were seen for peak force, peak stress, energy uptake or stiffness between fresh samples and samples stored for 24 h at 8 °C in the fridge or after 7 days at − 18 °C in the freezer (Table 1). However, estimation of elastic modulus was increased by 48% after 1 week of storage in the freezer (p = 0.048), when compared with the fresh samples. Furthermore, when compared to the fresh samples, gap length was longer in the frozen samples (p < 0.001), while transverse area was larger in the refrigerated samples (p = 0.024).

**Table 1:** Mechanical results from fresh samples and samples stored in the fridge or freezer.

	Fresh (mean ± SD)	Fridge (mean ± SD)	Freezer (mean ± SD)	Anova *P* value
Pull-to-failure test (exp. 1)				
Transverse area, mm^2^	15.6 ± 2.4	18.8 ± 3.2*	15.9 ± 2.3	**0.022**
Callus width, mm	5.9 ± 0.5	6.3 ± 0.5	5.9 ± 0.6	0.108
Callus depth, mm	3.4 ± 0.3	3.8 ± 0.5	3.5 ± 0.4	0.098
Gap length, mm	8.3 ± 0.5	8.3 ± 0.8	9.8 ± 1.0*	**0.001**
Peak force, N	32.9 ± 8.0	36.7 ± 4.4	32.1 ± 8.4	0.323
Stiffness, N/mm	3.2 ± 1.4	3.2 ± 1.4	4.2 ± 1.5	0.273
Peak stress, MPa	2.1 ± 0.5	2.0 ± 0.4	2.0 ± 0.5	0.824
Est. of elastic modulus, MPa	1.7 ± 0.7	1.5 ± 0.8	2.6 ± 0.8*	**0.013**
Energy uptake, N/mm	80 ± 23	92 ± 16	80 ± 21	0.286
Creep test (exp. 2)				
Transverse area, mm^2^	17.0 ± 4.0	19.5 ± 1.3	19.0 ± 3.0	0.156
Gap length, mm	10.4 ± 1.0	9.3 ± 0.6*	10.3 ± 1.1	**0.021**
Creep %	1.5 ± 0.1	1.6 ± 0.3	1.5 ± 0.3	0.458
Elongation up to 8 N, mm	2.1 ± 0.4	2.5 ± 0.6	2.6 ± 0.5	0.104
Water content % of ww	85.2 ± 1.1	85.5 ± 1.1	87.5 ± 1.0*	** < 0.001**

Pull-to-failure test was analyzed in experiment 1 and creep test was analyzed in experiment 2 (n = 10). Est. of elastic modulus means estimation of elastic modulus. SD = standard deviation. Bold means significant a difference.

*Significantly different compared to fresh samples. % of ww indicates percentage of wet weight.

### The effect of storage on creep, water content and structural properties (experiment 2)

**Figure 1:**
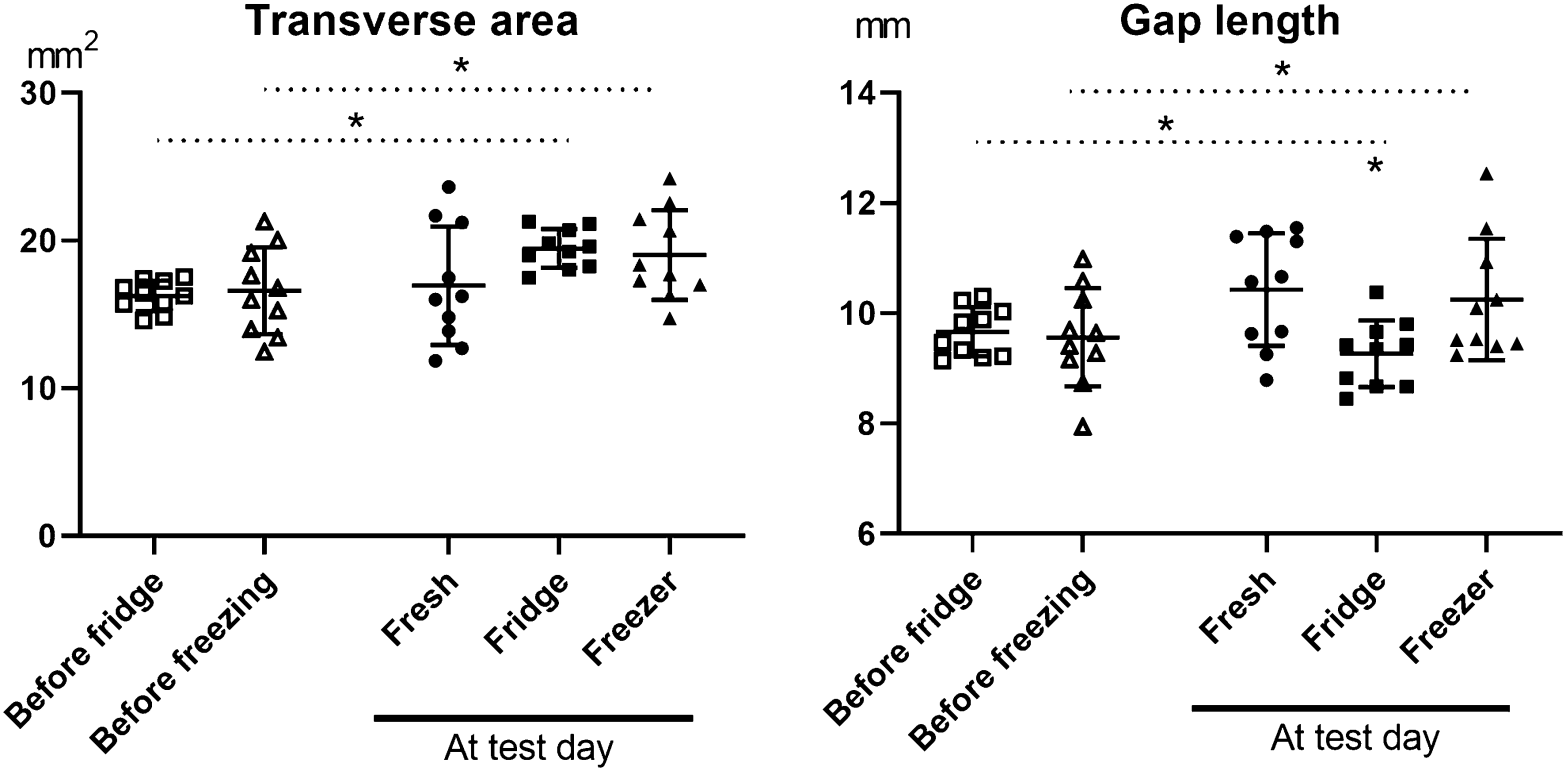
Transverse area and gap length measurements from experiment 2. Measurements were done before samples were stored in the fridge or freezer and after storage, at the mechanical testing day (n = 10). The line represents the mean and the bars SD. Image created using GraphPad Prism software, version 8. https://www.graphpad.com/scientific-software/prism/.

Viscoelastic properties, here measured by creep, were not altered by storage (Table 1). In contrast to experiment 1, there was no statistical difference in transverse area between the three groups, while gap length was decreased in the refrigerated samples compared to the fresh (p = 0.018). However, experiment 2 included structural measurements of the samples both before they were stored, and after storage, at the day of mechanical testing. Paired t-tests between these measurements showed an increased transverse area after storage, irrespective of temperature (fridge or freezer, p < 0.001 for both, Fig. 1). There was also an increased gap length between the pre and post storage measurement in the frozen samples but a decreased gap length in the refrigerated samples (p = 0.02 for both). There was a clear correlation between the pre and post measurement in the transverse area for the fresh (R^2^ = 0.949) and the frozen (R^2^ = 0.866) samples (p < 0.001 for both) but not for the refrigerated samples (R^2^ = − 0.099, p = 0.393). A significant correlation between the measurements of the gap length was seen in all groups (p < 0.02). The water content was increased after storage (p < 0.001; Table 1), primarily due to increased levels in the frozen samples (p < 0.001) but not in the refrigerated samples (p = 0.087).

### The effect of preconditioning in a pull-to-failure test (experiment 1 and 2)

**Figure 2:**
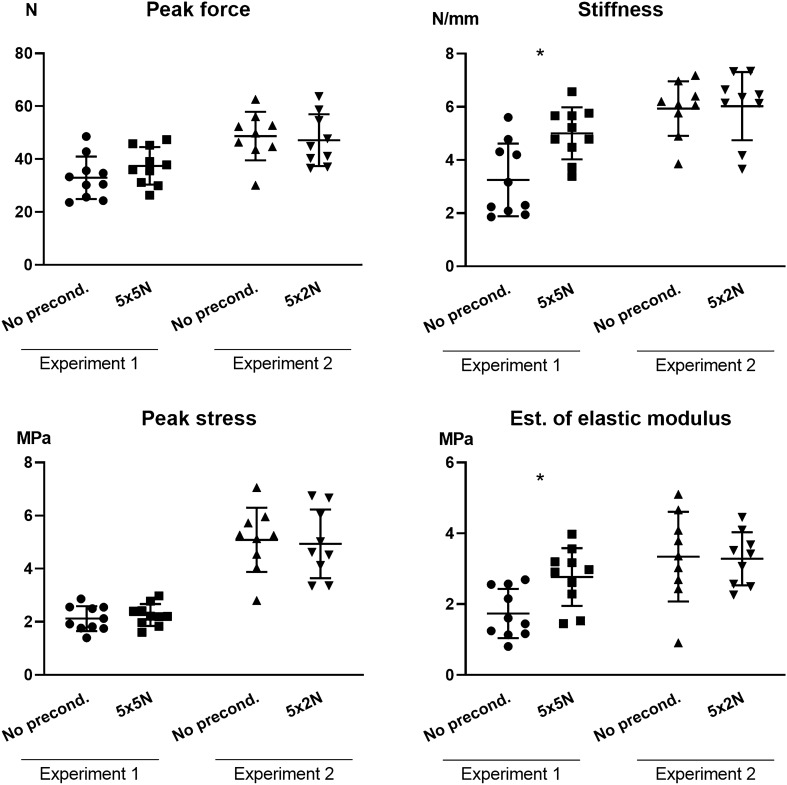
Mechanical results from samples with or without preconditioning. Preconditioning in experiment 1 was performed with 5 cycles up to 5 N (n = 10) and preconditioning in experiment 2 was performed with 5 cycles up to 2 N (n = 9). The preconditioned samples were compared to control samples without any preconditioning. N = Newton; x = cycles. The line represents the mean and the bars SD. Image created using GraphPad Prism software, version 8. https://www.graphpad.com/scientific-software/prism/.

A high level of preconditioning with 5 cycles up to 5 N (Experiment 1) did not affect the peak force or the peak stress, but it resulted in an increased stiffness and estimated elastic modulus by 50–60% (p ≤ 0.007) compared to samples without any preconditioning (Fig. 2). A lower level of preconditioning with 5 cycles up to 2 N (Experiment 2) did not lead to any altered structural or material properties (Fig. 2).

### The effect of storage on tissue morphology and cell viability

**Figure 3:**
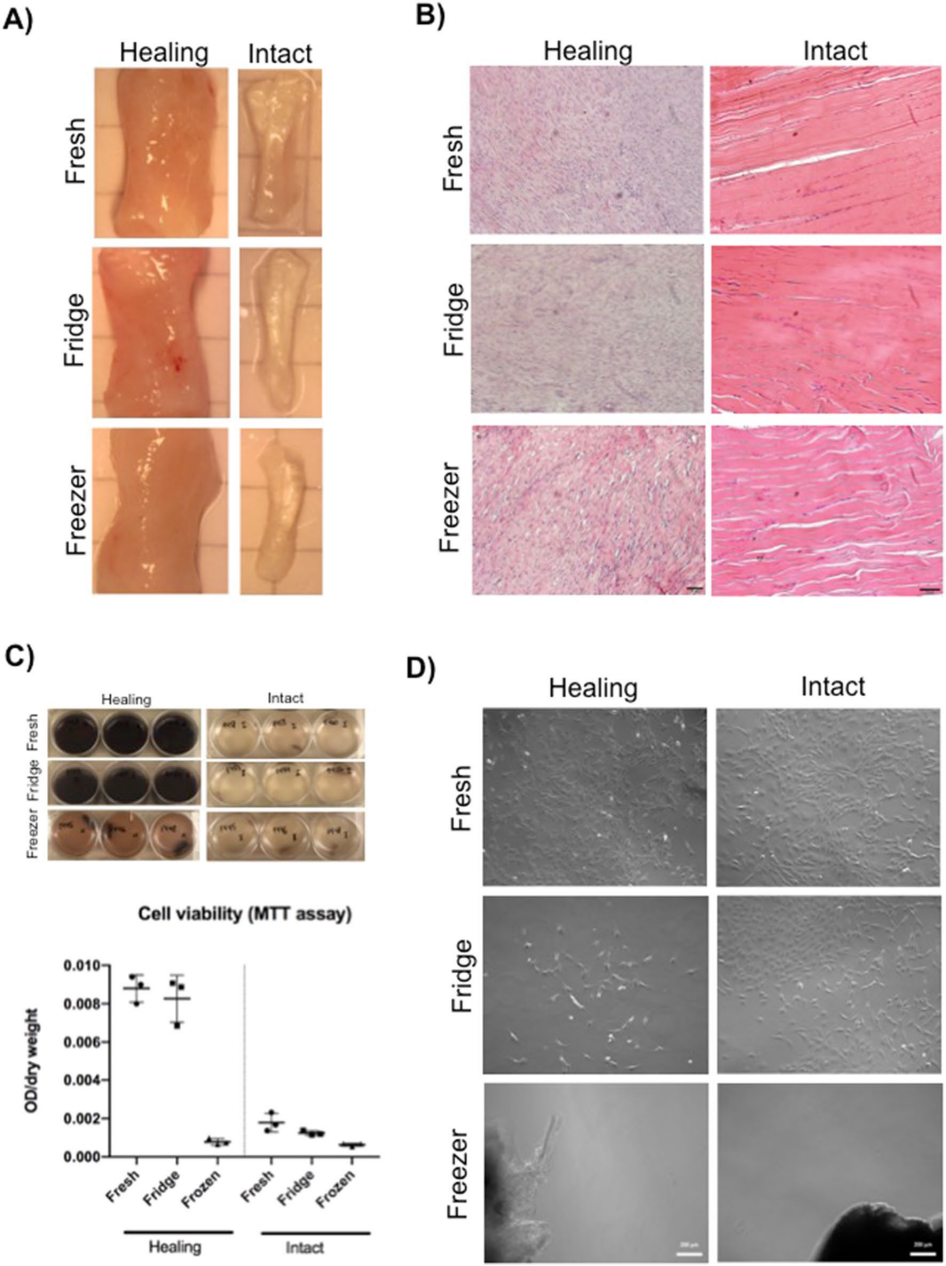
Tissue morphology and cell viability. (**A**, **B**) Macroscopic and microscopic images from intact and healing tendons after storage. Microscopic images are stained with hematoxylin–eosin. Scale bar for intact tendons 50 µM and healing tendons 20 µM. (**C**) Images of 6-well plates after the dissolvement of formazan crystals in DMSO. Graph represents the MTT assay on intact and healing tendons, verified by absorbance (OD) measurements. The OD is normalized against tissue dry weight. The line represents the mean and the bars SD. (**D**) Explant culture with intact and healing tendons after two weeks. Notice the explants in the frozen samples, where no fibroblast growth was verified. Scale bar 200 µM. Images created by using the software cellsSens Entry (version 1.8.1, Olympus Corporation, www.olympus-sis.com), Dino Capture 2.0 (version 1.5.3.4, AnMO Eletronics Corporation, www.dino-lite.com), Zen 2 (blue edition, version 2.0.0.1, Zeiss, www.zeiss.com) and GraphPad Prism (version 8, https://www.graphpad.com/scientific-software/prism/).

Hematoxylin–Eosin staining showed regional variations in the healing tendons. There was overall more spacing between collagen fibers in both intact and healing tendons after storage in the freezer in comparison to fresh and refrigerated ones. Albeit, the spacing was more distinguishable in the intact tendons. Furthermore, the tissue appeared somewhat more disorganized in the refrigerated healing tendon compared to the fresh tendon. Both the MTT assay and the explant model demonstrated more live cells in fresh and refrigerated healing tendons compared to frozen tendons. The pattern was similar for the intact tendons, but with less intensity in the MTT assay due to less cells (Fig. 3).

## Discussion

Biomechanical testing is an important resource used to analyze structural and material properties of different connective tissues. In this study, we compared if fresh healing rat Achilles tendons (tested straight after harvest) showed different mechanical proprieties compared to samples that have been stored either in the fridge (for 24 h) or in the freezer (for 1 week). Our results showed that peak force, peak stress, energy uptake, stiffness, and creep were all insensitive to storage. However, structural properties such as transverse area and gap length could indeed be altered by storage mainly through an increased water influx. These findings suggest that samples can be stored before mechanical testing, but measurements of structural properties should preferably be done at the day of testing, under equal conditions.

Our results are partly in line with previous studies on intact tendons. Studies using a single freeze–thaw cycle have also shown unaltered mechanical parameters when performing pull-to-failure or stress-relaxation tests^8,14,15^. On the other hand, it has been reported that the ultimate tensile failure can be altered in intact tendons after storage in the freezer^7^, and that repeated freeze/thaw cycles can lead to a reduced maximum load, maximum stress, and changes in collagen fibers alignment^6^. We observed a 48% increase in elastic modulus after the samples were stored in the freezer. This both contrast and corroborate with results from intact tendons where static or dynamic modulus have been shown to be either reduced or increased after a single or multiple freeze–thaw cycles^7,14^. Our measurements of static elastic modulus was done by normalization of the stiffness against transverse area and gap length, which were both altered by freezing. Taken together this suggests that elastic modulus is sensitive to freezing temperatures. Hence, the storage of samples should be chosen carefully when the elastic modulus is the main interest. Furthermore, healing tissues are morphologically different from the intact tendons, as seen in our macroscopic and microscopic images. Intact tendons contain a highly organized dense extracellular matrix, mainly composed of collagen type I and few cells^16^. On the other hand, injured tendons contain a disorganized loose connective tissue, with a higher water content, and show hypercellularity^12^. The tissue heals through formation of a fibrous scar tissue that forms a huge callus. The callus tissue will decrease in size over time when the matrix is replaced with a more functionally material^4,5^. The difference in extracellular matrix composition, cell number, and water content in these tissues could explain the differences between our study and previous ones.

An increased transverse area was found after storage in both the refrigerated and frozen samples compared to the fresh. Upon storage in either the fridge or freezer, samples were wrapped in gauze containing saline solution, and frozen samples were slowly thawed in the fridge overnight prior to the mechanical testing. This could explain why also the frozen samples absorbed more water and increased in size. In fact, frozen samples had significantly higher water content compared to fresh samples. Albeit not significant, the refrigerated samples also contained more water compared to fresh. The increased water content was also visualized in the microscopical images by spacing between the collagen fibers. An increased water content might be related to an larger extracellular area due to cell shrinkage, and this effect has been shown to be correlated with freezing time rather than temperature^17^. The storage time might therefore be an important factor to take into consideration. The time-points chosen in this study regarding storage condition were based on laboratory routines. When the use of fresh samples is unworkable, samples are instead stored for a maximum of 24 h in the fridge or 1 week in the freezer, and this can still be considered as a short-term storage. A prolonged storage time could potentially have more deleterious effects. Intact Achilles tendons respond differently to short or long-term storage in the freezer: long period (9 months) and repeatedly thawing (up to 5) lead to tissue deterioration while short periods (up to 3 months) and once or twice thawing do not have major effects on mechanical properties^15^.

The effect of long-term storage on healing tendons has not been studied here and might be important to evaluate in the future. Furthermore, we used pull-to-failure test or creep test while performing this study. The use of stress-relaxation tests or frequency sweeps in a dynamic protocol could reveal changes in for examples dynamic modulus or changes at different strain levels. Such protocol would provide a wider view of the viscoelastic properties of the healing tendon after different storage conditions and preconditioning levels. A previous study using a dynamic protocol on intact tendons showed changes in dynamic Young’s modulus after repeated freeze–thaw cycles, while no change in static Young’s modulus was observed^14^. However, healing tendons are weaker than intact tendons and the use of prolonged dynamic protocols could be complex. It has been shown that prolonged protocols on healing tendons can result in tendon failure before the final pull-to-failure test^18^. Another aspect to consider with healing tendons is the regional variation in matrix organization as seen in the histological evaluation in this study as well as in other studies^18,19^. Mechanical evaluation on regional differences in tendon properties might reveal even more insights in the effect of storage and preconditioning in the future.

The transverse area in experiment 1 was measured on the day of mechanical testing, hence, at different days for each group as all surgeries were performed on the same day. The different time-points of sample measurements can be considered as a limitation of this study. Therefore, in the second experiment, we performed the surgeries on different days to allow measurement and testing of all samples on the same day. To further clarify how the healing tendon tissue is affected by storage regarding structural properties, each sample was measured twice, first prior to the storage and thereafter straight before mechanical testing. Experiment 1 showed an increased transverse area in the fridge-stored samples but not in the frozen samples while experiment 2 showed no significant difference between the groups on the day of testing. However, paired t-tests of the measurements pre and post storage showed an increased transverse area after both storage conditions. On the other hand, we also observed that the gap length was affected differently depending on the storage condition in both experiment 1 and 2. Samples stored in the fridge had a decreased gap length whereas freezing increased the length. The changes in gap length might be linked to cell-mediated effects. Freezing tissues without cell protective actions leads to cell degradation as shown in frozen tendons and ligaments^14,20^ while short term storage at 4 °C normally protects cells. Our findings with the MTT assay and explant culture corroborate that cell viability is preserved when tendons are stored in the fridge but not in the freezer. Tendon cells in vitro can pull on the unloaded matrix^21,22^. Our tendons in the fridge are unloaded and therefore possibly subjected to cell-mediated extracellular matrix contraction during the storage time. This could perhaps explain the shorter gap length finding as well as the more disorganized appearance in the healing matrix after fridge storage compared to fresh and frozen samples. This contrast to the cells from frozen samples that die^14^, and possible instead release their tension to the extracellular matrix and perhaps have a disrupted cell-to-cell connection, which could explain the increased gap length. Frozen explants did not proliferate when cultured for 2 weeks, whereas refrigerated and fresh samples showed cell growth. Besides, frozen tissues also form ice crystals and the total tissue volume cannot contract due to this. Furthermore, sample storage in the freezer can trigger changes in morphological appearance, and together with the osmosis process, result in mechanical damage to the cellular structures^17^.

Another important finding with our study was the diverse response between the preconditioning levels in healing Achilles tendons. Five cycles up to 5 N lead to significantly stiffer tendons while five cycles up to 2 N showed no difference between the groups. This demonstrates the importance of knowledge on tissue strength prior to preconditioning execution. This might also limit the use of preconditioning in experiments where groups differ greatly in total strength, as the same protocol could potentially result in different strain levels. The strength of the healing tissue differs more compared to intact tendons depending on the treatment, such as loading conditions, drug exposure and the length of the healing period used in each study^1,2,13,23,24^. The 5 N of preconditioning corresponded to ~ 15% of the maximum force of the healing tendon, while 2 N corresponded to ~ 4% of the maximum force. The lack of an overall and a specific effect on peak force when using low preconditioning on healing tendons contrast to what has previously been shown on intact tendons. Preconditioning performed at ~ 1–3% of the ultimate failure load on intact tendons led to increased ultimate failure load and stress^25^.

In conclusion, there were no major effects on the peak force, stress, or viscoelastic properties of the healing tendon after different storage conditions or preconditioning levels. However, freezer storage has a major effect on cell viability, in contrast to fridge storage or fresh samples. Notwithstanding, different storage conditions should be carefully considered when elastic modulus, water content, transverse area, or gap length are the primary outcomes. It is also important to consider the strength of different groups (e.g. different treatment groups) when preconditioning is used. If the strength differs too much between groups, one might encounter unspecific differences due to the preconditioning level. Furthermore, it is essential to store all samples in an equivalent way and to perform measurements of structural properties (e.g. transverse area and length) on the day of mechanical testing and not straight after tissue collection.

## Methods

### Study design

In total, 103 female specific-pathogen free (SPF) Sprague–Dawley rats 10–11 weeks old (Janvier, Le Genest-Saint-Isle, France), weighing on average 252 g (SD20) were used in three separate experiments to study how elastic and viscoelastic properties of healing Achilles tendon are affected by different storage conditions and preconditioning levels.

#### Experiment 1

**Figure 4:**
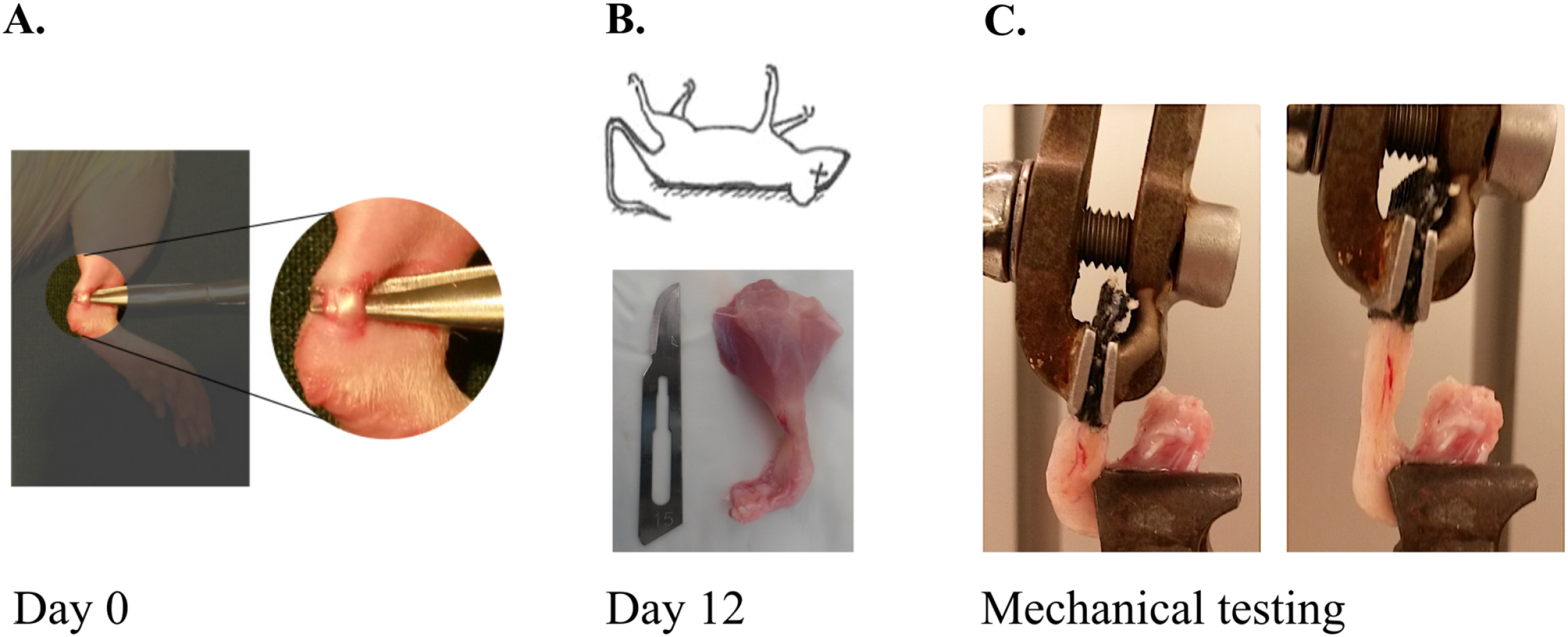
Experimental setup. (**A**) On day 0, a transversally skin incision was performed on the right hind limb and the tendon complex was exposed. The plantaris tendon was removed and the Achilles tendon was transected. (**B**) Animals were euthanized and tissue samples were collected 12 days post-surgery. (**C**) Samples were fixed in a metal clamp in the material-testing machine. Mechanical testing was performed according to each group.

**Figure 5:**
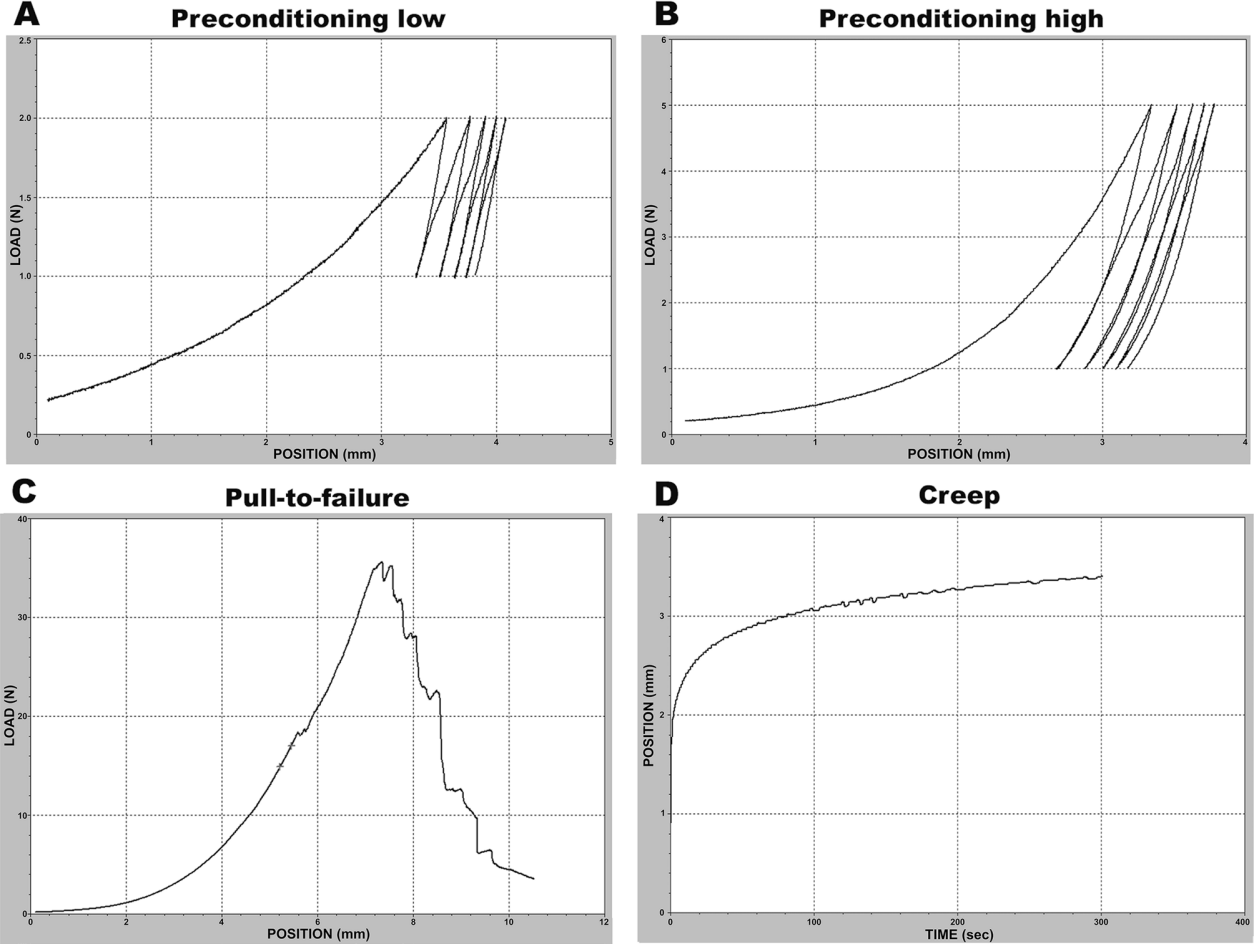
Representative overview of the type of mechanical test and preconditioning level performed in each experiment. Graphs are from a fresh sample. (**A**, **B**) represents the preconditioning graph, where 5 cycles of 2 N (**A**) or 5 N (**B**) were applied. (**C**) Pull-to-failure test. (**D**) Creep test with a constant load of 8 N for 300 s. Graphs were obtained from the mechanical testing (100R; DDL INC., EDEN PRAIRIE, MN) software MtestW, version5.1.0 (ADMET). https://www.admet.com.

Achilles tendon transection was performed on 40 rats (all on the same day) and the animals were randomized to 4 different groups; Fresh/No preconditioning, Fridge, Frozen and Preconditioning high (Table 2). Sample collection was performed 12 days post-surgery (Fig. 4) and followed by mechanical testing (Fig. 5). Before the samples were subjected to a pull-to-failure test, the refrigerated samples were stored for 24 h at 8 °C and the frozen samples were stored for 7 days at − 18 °C. This test was therefore performed on different days for each group. The fresh, refrigerated, and frozen samples were all tested by a pull-to-failure test without preconditioning.

**Table 2:** Study design for mechanical measurements.

	Groups	Research questions		Precond 5 × 5 N (high)	Precond 5 × 2 N (low)	Pull-to-failure test	Creep test
Exp. 1	Fresh/precond high n = 10	Are elastic properties affected by high precond.?		×		×	
	Fresh/no precond n = 10		Are elastic properties affected by storage?			×	
	Fridge n = 10					×	
	Frozen n = 10					×	
Exp. 2	Fresh/precond low n = 9	Are elastic properties affected by low precond.?			×	×	
	Fresh/no precond n = 9					×	
	Fresh n = 10		Are the viscoelastic properties affected by storage?		×		×
	Fridge n = 10				×		x
	Frozen n = 10				×		×

*Precond.* preconditioning; × means that the procedure was executed.

In the preconditioned high group, samples were fresh and subjected to 5 cycles of 5 N of preconditioning before the pull-to-failure test. This group was thereafter compared to the fresh samples with no preconditioning.

#### Experiment 2

48 rats were randomized to 5 different groups prior to the surgery; fresh, fridge, frozen, preconditioning low and no preconditioning. In contrast to experiment 1 (where we saw structural changes in the stored tendons), the Achilles tendon transection surgery was executed on different days to allow mechanical testing on the same day for all samples. Fresh, refrigerated, and frozen samples (n = 10/group) were all pre-conditioned with 5 cycles up to 2 N prior to a creep test. The transverse area and gap length were measured twice in experiment 2, straight after tissue collection and after storage (on the day of mechanical testing). The water content from the healing tendons was also measured in experiment 2, after the mechanical test. The two groups: preconditioning low and no preconditioning (n = 9/group) were subjected to a pull-to-failure test as in experiment 1, with or without a lower level of preconditioning of 5 cycles up to 2 N (as high preconditioning had an effect on stiffness and modulus in experiment 1).

#### Experiment 3

15 rats were randomized into three groups: fresh, fridge and frozen. Rat Achilles tendon was transected on different days and stored as the previous experiments. This enable data collection at the same time point. Both healing and intact tendons were used for histology (n = 1), MTT assay (n = 3), and explant culture (n = 1).

### Animals and housing

The animals were kept in acrylic cages, in pairs, and placed on ventilated racks under controlled humidity (55%) and temperature (22 °C) with the light–dark cycle of 12 h each. Standard food pellets and water were given ad libitum. All cages were provided with shredded paper, wooden pegs and hiding places. The experiments adhered to the institutional guidelines for the care and treatment of laboratory animals. The Regional Ethics Committee for animal experiments in Linköping, Sweden, approved all the experiments (ID1424).

### Tendon injury

All animals were anesthetized with isoflurane gas and laid in a prone position. The skin on the right leg was shaved and cleaned with chlorhexidine ethanol. Subcutaneously antibiotic injection was given once preoperatively (25 mg/kg oxytetracycline) and analgesics injections were given pre- and postoperatively every 8–12 h for a period of 48 h (0.045 mg/kg buprenorphine, subcutaneously). The surgery day was counted as day 0. A small skin incision, lateral to the Achilles tendon was made and the tendon complex was exposed (Fig. 4A). The plantaris tendon was removed and the Achilles tendon was cut transversely in the middle part, using a no.15 surgical blade mounted in a scalpel, and left unsutured. The skin was closed with 2 stitches using a 4/0 non-absorbable monofilament suture (DAFILON). This procedure was performed under aseptic conditions and has been described elsewhere^1,2^.

### Sample collection and storage conditions

Twelve days after the surgery, the rats were euthanized with carbon dioxide and samples were collected (Fig. 4B). The right Achilles tendon, together with the calcaneal bone and calf muscle, was harvested and placed in gauze with 0.9% saline solution. The sagittal and transverse diameter of the midpart of the callus tissue was measured immediately after collection using a slide caliper, and the transverse area was calculated by assuming an elliptical geometry. The distance between the old tendon stumps was visualized through transillumination and the gap length was measured by a caliper. Fresh samples were tested mechanically the same day as the animals were euthanized. The remaining samples were either stored in the fridge (8 °C) for 24 h or in the freezer (− 18 °C) for 1  week. Frozen samples were thawed slowly, overnight in the fridge, before mechanical testing performance.

### Mechanical testing

The major part of the muscle tissue was removed from the tendon, and the proximal part of the tendon was fixed in a metal clamp between two pieces of sandpapers. The calcaneal bone was fixed in a custom-made clamp at 30° dorsiflexion relative to the direction of traction in the material-testing machine (100R; DDL INC., EDEN PRAIRIE, MN) (Fig. 4C).

#### Preconditioning

Ten samples from experiment 1 and 38 samples from experiment 2 were preconditioned prior to the data recording test (pull-to-failure or creep test, Fig. 5). The preconditioning high group in experiment 1 was subjected to 5 cycles of 5 N (Fig. 5B) at a speed of 0.1 mm/s or and the preconditioning low group was subjected to 5 cycles of 2 N using the same speed (Fig. 5A). All groups subjected to the creep test in experiment 2 (fresh, fridge, and frozen samples) were preconditioned before the creep test (5 cycles of 2 N, Fig. 5A).

#### Pull-to-failure test

Samples were pulled at a constant speed of 0.1 mm/s until failure (Fig. 5C). Peak force at failure (N) and energy uptake until failure (N/mm) were measured by the machine software (MtestW, version5.1.0, ADMET). A linear portion of the elastic phase of the curve was marked by the investigator, for automated stiffness (N/mm) calculation by the machine software. Peak stress (MPa) and estimate of elastic modulus (MPa) were calculated assuming an elliptical cylindrical shape and homogeneous mechanical properties. Estimation of elastic modulus was calculated as stiffness × gap length/transverse area and peak stress as peak force/transverse area^1,2^.

#### Creep test

Samples were pulled to 8 N (2 mm/s) and thereafter kept under a constant load of 8 N for 300 s (Fig. 5D). The viscoelastic properties of the healing tendons were measured through the progressive deformation of the tissue and calculated as a percentage of the starting length.

### MTT (colorimetric) assay

A MTT assay was used for quantification of cell viability after storage. The tendons were incubated for 3 h in DMEM/F-12 without phenol red (gibco) with 0.5 mg/mL MTT solution (3-4,5dimethylthiazol-2yl)-2,5-diphenyltetrazolium bromide), on an orbital shaker protected from light. The tendons were thereafter transferred to 7 mL DMSO and kept 1hour on an orbital shaker, protected from light. Purple formazan crystals were dissolved and 100 μL DMSO solution was used for absorbance reading. Absorbance was quantified at 550 nm with 680 nm as the reference wavelength. The darker the solution, the higher the number of active/viable cells. The absorbance value (OD) was normalized to the tissue dry weight.

### Explant culture

Intact and healing tendons after storage were cleaned in PBS containing 1% penicillin–streptomycin (10.000 U/mL, gibco). Samples were cut into small pieces and placed in 25 cm^2^ cell culture flasks. Explants were kept for two weeks in culture with DMEM/F-12 containing l-glutamine, no phenol red and hepes (gibco), supplemented with 10% FBS (fetal bovine serum, heat inactivated, South American origin, gibco) and 1% penicillin–streptomycin (10.000 U/mL, gibco). After two weeks, fibroblast growth was confirmed by using a microscope (Axio Vert.A1 fluorescence microscope Carl Zeiss) with an N‐Achroplan 10 ×/0.25 objective (Carl Zeiss) and Axiocam 503 color camera (Carl Zeiss) Zen 2 (blue edition, version 2.0.0.1, Zeiss).

### Histology

Intact and healing tendons were stored accordingly and thereafter fixed in 4% phosphate-buffered formaldehyde overnight, followed by dehydration and paraffin embedding (n = 1 for each group: fresh, refrigerated and frozen). Longitudinal sections (7 µM) were made and haematoxylin–eosin (HE) staining was performed. Images were captured using a light microscope (Olympus BX51) with an attached camera (Olympus DP73) and the software cellsSens (Entry version 1.8.1, Olympus Corporation). An UPlanFI 10 ×/0.30 objective lens was used for the healing tendons, while 20 ×/0.50 objective lens was used for the intact ones.

### Water content measurement

Subsequently to the mechanical test performed in experiment 2, samples were weighted and then dried in a freeze-drying chamber (SAVANT, FDC206) for 24 h. Afterward, samples were weighted once more and the water content of each sample was calculated and normalized to the initial wet weight (%).

### Statistics

GraphPad Prism software version 8 was used for data analyses. The primary variable outcome for the pull-to-failure and preconditioning test was peak force. For the creep test, the viscoelastic propriety of the healing connective tissue was considered the primary outcome. A one-way ANOVA was used to analyze differences between the three storage conditions (fresh, fridge or freezer) followed by a Dunnett’s multiple comparisons test where the two storage groups were compared to the fresh control group. A student’s t-test for independent samples was used to analyze the effect of preconditioning on fresh samples. A paired t-test and paired sample correlation analysis were used to investigate the measurements differences from before and after storage conditions (fridge and freezer) for transverse area and gap length.

## Data Availability

The datasets generated during and/or analyzed during the current study are available from the corresponding author on reasonable request.
